# Perspectives of healthcare professionals on medical care in nursing homes in Germany and The Netherlands: an explorative study using qualitative content analysis

**DOI:** 10.1093/eurpub/ckaf176

**Published:** 2025-09-28

**Authors:** Alexander M Fassmer, Adele Grenz, Markus Ennen, Sytse U Zuidema, Kathrin Boerner, Sarah I M Janus, Yvet Mooiweer, Falk Hoffmann

**Affiliations:** Department of Health Services Research, Division of Outpatient Care and Pharmacoepidemiology, Carl von Ossietzky Universität Oldenburg, Oldenburg, Germany; Department of Health Services Research, Division for Prevention and Rehabilitation Research, Carl von Ossietzky Universität Oldenburg, Oldenburg, Germany; Department of Health Services Research, Division of General Practice/Family Medicine, Carl von Ossietzky Universität Oldenburg, Oldenburg, Germany; Department of Primary and Long-Term Care, University Medical Center Groningen, University of Groningen, Groningen, The Netherlands; Department of Health Services Research, Division for Prevention and Rehabilitation Research, Carl von Ossietzky Universität Oldenburg, Oldenburg, Germany; Department of Primary and Long-Term Care, University Medical Center Groningen, University of Groningen, Groningen, The Netherlands; Department of Health Services Research, Carl von Ossietzky Universität Oldenburg, Oldenburg, Germany; Department of Orthopedics, University of Groningen, University Medical Center Groningen, Groningen, The Netherlands; Department of Health Services Research, Division of Outpatient Care and Pharmacoepidemiology, Carl von Ossietzky Universität Oldenburg, Oldenburg, Germany

## Abstract

The organization of healthcare for nursing home residents varies widely between systems, even between neighbouring countries such as Germany and the Netherlands. This study compares healthcare professionals’ perspectives on strengths and challenges in medical care for nursing home residents in Germany and the Netherlands. Semistructured interviews were conducted in Germany with six nursing staff from six nursing homes and six general practitioners (GPs) in private practice and in the Netherlands with one elderly care physician (ECP) and seven nursing staff members from six nursing homes between August 2023 and March 2024. Interviews were audio recorded, transcribed, translated, and analysed using qualitative content analysis. Participants reported that Germany and the Netherlands face rising long-term care demands due to aging populations, however, their nursing home care models differ substantially. In Germany, care is reactive and fragmented, with external professionals, especially GPs, providing care. Challenges include delays, limited communication, and a lack of standardized processes. Conversely, the Netherlands adopts a structured, preventive approach, led by ECPs supported by multidisciplinary teams. This model emphasizes proactive monitoring, team collaboration, and holistic care but faces workload challenges and limited specialist access. Interprofessional collaboration is more hierarchical and record-based in Germany, while it is team-oriented and conversational in the Netherlands. This study highlights key differences in the organization of nursing home care in Germany and the Netherlands, particularly in access to specialists, interprofessional collaboration, and structures. Potential adaptations to improve care must fit within the existing structures of each healthcare system.

## Introduction

Nursing home residents often live with multiple chronic conditions, such as cardiovascular diseases [1], frailty [2], cognitive and functional impairment [3], and polypharmacy [4]. These needs pose challenges for care delivery, especially regarding coordination [5, 6]. Ensuring high-quality medical care is a central concern in long-term care across many European healthcare systems [7]. However, medical care provision varies considerably—e.g. between Germany and the Netherlands [8]. These shared challenges are addressed through nationally specific approaches to organizing and financing long-term care, which shape the conditions under which medical care is delivered [9, 10].

In Germany, around 800 000 residents (1.0% of the population [11]) receive care from general practitioners (GPs) in private practice. Patients can choose their own physicians [12], and may retain their GP if located nearby. Care is often delivered by multiple GP practices [13]. Specialist care is provided externally.

In the Netherlands, care settings range from small-scale, home-like residential settings (e.g. formerly verzorgingshuizen) to larger institutional nursing homes (Table 1). The prototypical model of Dutch nursing home care is delivered by elderly care physicians (ECPs)—a distinct specialty focused on frailty. Approximately 122 000 residents (0.7% of the population [14]) receive care from ECPs employed directly by long-term care organizations. ECPs lead multidisciplinary teams including nurses, physiotherapists, and others [15]. Specialist care is available only via hospital or polyclinic referral.

**Table 1.** Comparative overview of nursing home organization and staffing

	Germany	The Netherlands
Legal basis and financing of long-term care	Statutory Long‑Term Care Insurance under the Social Code Book XI (SGB XI): funded by membership contributions; covers only parts of costs incurred, remainder is covered by private co-payment or—in case of social need—by social welfare [32].	Long‑Term Care Act (Wet langdurige zorg, Wlz) (in force since 2015) as the core scheme for institutional long‑term care; funded by general income tax [33]; when claiming long-term care benefits, individuals have nationally uniform co-payments (amount depends on sociodemographic factors and care setting [34]).
Facility types	Nursing homes (NHs, Pflegeheime).	Two main settings: (1) Nursing homes (NHs, verpleeghuizen) providing high‑dependency long-term care with on‑site medical care. (2) Small‑scale home‑like facilities and former residential homes (SHNHs, verzorgingshuizen) where medical care is typically provided by community GPs.
Nursing home population	800 000 residents (2023 [11]).	(1) NHs: 122445 residents (2023 [14]). (2) SHNHs: Resident counts included in Wlz totals but not always reported separately.
Organization of medical care in facilities	Medical care delivered predominantly by off-site community GPs (Hausärzte/Allgemein mediziner) and other specialists in private practice who visit the NHs; facilities generally do not employ physicians.	(1) NHs: medical care is led by on-site ECPs (Specialisten Ouderengeneeskunde) employed by or contracted to the respective NH organization; embedded in multidisciplinary teams. (2) SHNHs: GPs provide primary medical care.
Registered nurses	Registered nurses (RNs, Gesundheits- und Krankenpfleger, Altenpfleger) responsible for nursing assessments, care planning, medication administration, and clinical supervision of assistant staff.	(1) NHs: Registered nurses (RNs, Verpleegkundigen) with roles in clinical leadership, complex care, and coordination of multidisciplinary teams. (2) SHNHs: RNs present but typically in smaller numbers; roles often combined with care coordination.
Assistant nurses/Nursing aides	Geriatric care assistants (Altenpflegehelfer) or health care assistants (Krankenpflegehelfer) supporting RNs in basic care.	(1) NHs: Verzorgenden IG (Individuele Gezondheidszorg, intermediate vocational trained assistant nurses) provide basic nursing and personal care under the supervision of RNs. (2) SHNHs: Comparable assistant nursing roles.
Nurse specialists/advanced practice nurses	Advanced Practice Nurses and Nurse Practitioners exist in pilot projects or specific institutional initiatives (e.g. wound care, dementia care), but they have no formally defined legal scope of practice comparable to the Netherlands.	(1) NHs: Nurse specialists (verpleegkundig specialisten) in many organizations, often focusing on chronic disease management and complex coordination. (2) SHNHs: Rare.
Allied health professionals	Physiotherapists, occupational therapists, speech and language therapists usually accessed via external providers on physician referral; dietitians and social workers involved in some facilities.	(1) NHs: Physiotherapists, occupational therapists, speech and language therapists, psychologists, dietitians, and social workers are commonly integrated into the in-house multidisciplinary team. (2) SHNHs: Allied health professionals mostly accessed via external providers on GP referral.

ECP, elderly care physician; GP, general practitioner; NHs, nursing homes; RN, registered nurse; SHNHs, small‑scale home‑like facilities and former residential homes; Wlz, wet langdurige zorg.

These structural differences influence health care utilization. For instance, German residents are hospitalized more often than Dutch residents [16]. A survey found ECPs in the Netherlands are more involved in hospitalization decisions than GPs in Germany [17], where staff also more frequently reported unnecessary transfers. Dutch staff reported higher satisfaction with medical care and staffing [18]. Typically, one ECP is responsible for a facility in the Netherlands [15], compared to an average of eight GP practices per facility in Germany [13]—complicating collaboration.

This study compares the perspectives of nursing staff and primary care physicians (GPs/ECPs) on nursing home medical care in Germany and the Netherlands with respect to perceived strengths and challenges.

## Methods

### Study design

This explorative study using qualitative content analysis [19] was embedded in the subproject “Medical care provision in nursing homes and its influence on residents’ health” of the study Comparison of healthcare structures, processes and outcomes in the Northern German and Dutch cross-border region I (CHARE-GD I) [18].

Ethical approval was obtained from the Carl von Ossietzky University of Oldenburg (No. 2023-152) and the University Medical Center Groningen (METc 2023/404). All participants provided written informed consent for the interviews, including recording, transcription, and analysis. For data exchange between study sites, a data transfer agreement for pseudonymized data was established.

### Participants and recruitment

The sample size was determined *a priori* based on feasibility, aiming for 24 participants—six nurses and six GPs/ECPs per country. Nurses were required to be actively involved in care, not just administration. German GPs had to provide care in at least one nursing home.

In Germany, participants were recruited in north-western Lower Saxony. Fourteen nursing homes were contacted to recruit six nurses. GPs were recruited via a quality circle and personal contacts of a co-author (M.E.). Recruitment goals were met.

In the Netherlands, recruitment was supported by the University Network for Elder Care (UNO-UMCG), a collaboration between UMCG and 20 long-term care providers in the north and east of the country. Participants were invited via the network’s newsletter. In total, one ECP and seven nurses from six homes participated, so recruitment goals were partially achieved. Participant characteristics are summarized in Table 2.

**Table 2.** Characteristics of the participants

	Germany (*N* = 12)	The Netherlands (*N* = 8)
Sex		
Female	4	7
Male	8	1
Age range (years)	25–62	25–60
Profession		
Elderly care physician	NA^b^	1
General practitioner^a^	6	NA
Nurse	6	7
Years of experience in the current position		
1–5	5	6
More than 5	7	2

^a^Including internists in general practice.

^b^NA, not applicable.

### The interview guideline

The semistructured interview guideline [20] was developed by a German-Dutch team. Interviews were conducted between August 2023 and March 2024. The guideline (Table 3) was used in both countries, with minor system-specific adaptations. Topics included residents’ medical needs, care organization, interprofessional communication, strengths, and weaknesses of the healthcare system, and perspectives on the other country’s approach. Additional subquestions were asked as needed. Demographic data (e.g. age, sex, work experience) were collected beforehand.

**Table 3.** Perspectives of nursing staff and GPs/ECPs on medical care of nursing home residents—interview guideline (key questions)

What is your assessment of the overall medical care needs of the nursing home residents in your care?
For nursing staff: Overall, how do you rate the competence of GPs/ECPs in caring for nursing home residents? For GPs/ECPs: Do you feel that your training and further education as a GP/ECP has prepared you well enough to care for nursing home residents?
For nursing staff: How are primary care services provided in your facility? For GPs/ECPs: How are primary care services provided for the residents in your care?
How do you perceive the cooperation between nursing care and GPs/ECPs?
How do you rate the communication and cooperation between all the different professionals involved in the care of nursing home residents? This includes nursing staff, general practitioners, other specialists and allied health professionals (e.g. physiotherapists).
How could collaboration be improved?
In the context of caring for nursing home residents, what aspects of the health care system are supportive and what aspects are not supportive of your work as a nurse/GP/ECP?
What do you know about the organization of medical care for nursing home residents in the Netherlands?
After a brief summary of the organization of care in the other country: What do you think of the Dutch/German system of medical care for nursing home residents?
In an ideal world, how do you think the residents of a nursing home would receive the best possible medical care?

### Conducting the interviews, data collection, and transcription

In Germany, all interviews were conducted by A.M.F. (native speaker). Nurses were interviewed in their nursing homes; GPs either in practice settings or at home. Interviews lasted on average 53 minutes. One German GP interviewed in this study is also a co-author (M.E.). He was included for professional expertise and only contributed at the manuscript stage, not to interview or analysis. In the Netherlands, Y.M. (native speaker) conducted all interviews in the nursing homes, with an average length of 46 minutes.

All interviews were audio recorded and transcribed verbatim by an external service, with identifiers removed. Dutch transcripts were translated into German and verified by A.M.F./A.G.

### Data analysis

Two researchers (A.M.F. and A.G.) analysed the data using Mayring’s structured content analysis [21] with MAXQDA Analytics Pro 24 [22]. Deductive codes were based on literature (e.g. Refs. 9, 11, and 17) and the interview guide, while inductive codes emerged during analysis. Both researchers coded two transcripts jointly before independently coding all data. Discrepancies were resolved through discussion. Codes were reviewed comparatively by country, and overarching themes were developed. Selected quotes were translated into English to illustrate findings.

## Results

The analysis resulted in three primary themes, each divided into subcategories (Table 4), that contrast Germany and the Netherlands.

**Table 4.** Overview of main und subcategories

	Germany *vs*. The Netherlands	
Care situation—responding to residents’ needs	Individual	Collective
	Reactive	Preventive
Organization of nursing home care	External	Internal
	Many physicians	One nursing home physician
Interprofessional collaboration	Delegating	Independent
	Record-based	Conversation-based

### Care situation—responding to perceived needs of residents

In both countries, nursing home care was characterized by heterogeneity in health conditions and needs perceived by nursing staff and physicians. Interviewees reported a wide range of impairments and levels of dependency.

#### Individual vs. collective

A striking difference concerned how diverse needs were addressed, with Germany relying on an individual approach and the Netherlands on a collective strategy.

For the German interview partners, the care of nursing home residents often relied on the individual initiative of nurses and physicians. The lack of a multidisciplinary approach forced staff to develop isolated solutions, making care dependent on personal effort. Communication issues also hampered care and forced staff to delay or improvise solutions. When taking on new residents, physicians emphasized their need for comprehensive information transfer: “Especially for those (residents) who can’t convey the information themselves (…), it would be important to hear directly from the other physician.” (Physician-G-1).

In the Netherlands, in contrast, healthcare staff experienced shared responsibility for the care of nursing home residents and characterized nursing home care as being clearly structured and collectively organized. Multidisciplinary teams worked in close collaboration to address the individual needs of residents. Such collaboration would distribute responsibility across multiple parties: “The important thing is to keep in touch and hold multidisciplinary meetings about the residents on a regular basis. This is a good way to stay connected” (Nurse-NL-3).

#### Reactive vs. preventive

While respondents described nursing home care in Germany as more reactive, the Dutch participants placed greater emphasis on a preventive approach.

Various German interview partners described the responses to the perceived needs of nursing home residents as reactive. Differences in how needs were identified and interpreted were also evident between professional groups and countries. In Germany, both physicians and nurses identified insufficiently qualified staff as a substantial challenge in recognizing diverse needs at an early stage, posing a substantial barrier to delivering quality care. The high number of hospital admissions among nursing home residents was also raised as an example of reactive care. In this context, the lack of advance directives is another issue: “The doctor and nurses are often not very familiar with the resident. And then, of course, the families are often not available because they live further away […] Nobody is really in charge.” (Physician-G-5).

By contrast, Dutch professionals emphasized prevention, focusing on avoiding end-of-life treatments if no longer beneficial: “Coming to a nursing home typically means having reached the final stretch of life. It is more important to make the person feel comfortable than to presume treatment at all costs” (Nurse-NL-1). Upon admission to the facility, residents and their families would participate in advance care planning discussions, while the care team implements measures to improve quality of life, such as promoting social interaction, providing prophylactic treatment for pressure sores, and offering nutritional counseling.

### Organization of nursing home care

In both countries, the GP (Germany) and ECP (Netherlands) assume a central role. Care requires expertise in multiple specialties. Interviews revealed differences in organization and quality of medical care.

#### External vs. internal

A difference between the two countries was observed in the respondents’ explanations of how they experienced the different organizational approaches.

German respondents noted substantial differences in care quality due to the nature of external physicians, especially GPs. While some GP structures involve regular oversight, others have sporadic visits, making them hard to reach: “Most doctors, especially those who are further away, are hard to reach. If you try to call, you’ll often find that you can’t get through. […] It sometimes takes a week before you even get a response, or you have to send a fax several times” (Nurse-G-1). Additionally, logistical challenges, such as transportation to specialists in rural areas and the need for family or GPs to intervene, would hinder care. The lack of integration between external physicians also sometimes led to a loss of information, complicating continuity of care.

In the Netherlands, respondents highlighted the positive aspects of the ECP and the multiprofessional care team. Continuous on-site monitoring was particularly appreciated, “because it enables you to react quickly” (Physician-NL-1). The central role of the ECP also ensured the coordination of treatments and the delivery of holistic care. However, the system is not without its drawbacks, including restricted access to specialists for complex cases and the high workload of the ECP, which can result in bottlenecks.

#### Many physicians vs. one nursing home physician

Another organizational difference concerned the number and type of responsible physicians.

In Germany, while GPs provide most care, specialists are also involved, which nurses saw as advantageous. However, they felt GPs may lack sufficient training for the complex needs of residents, and fragmented care leads to communication, coordination, and referral problems. For a German GP, “optimal care of nursing home residents means clear responsibilities. One person must be in charge. […] This can only be a GP. And it would be desirable if the facility’s care protocols and regulations (i.e. who is the assigned physician and how is the personal autonomy of residents preserved) are discussed at the time of each new admission to a nursing home” (Physician-G-2).

In contrast, the Dutch ECP oversees comprehensive care and consults external specialists as needed. The Dutch participants thought that such a care model results in a strong doctor-patient relationship. The Dutch ECP explained that their role is to “navigate through the complex field and, above all, looks at the big picture rather than examining each health condition and impairment by itself” (Physician-NL-1).

### Interprofessional collaboration

Interprofessional collaboration was seen as essential, but differed between countries in structure, intensity, and professional roles.

#### Delegating vs. independent

Interprofessional collaboration in Germany was considered by respondents as more hierarchical, whereas in the Netherlands it was experienced as more team-oriented and inclusive, with an emphasis on shared decision-making.

In Germany, interprofessional collaboration is highly hierarchical, with physicians often dominating and delegating tasks to nurses or therapists. While this structure supports quick decision-making, it limits the autonomy of other professionals and reduces direct communication:” Whenever the physicians are here […], it’s fine. Then you can talk to them easily. But you also notice that they are always under a lot of time pressure. So, they often make decisions solely based on residents’ records.” (Nurse-G-1).

In the Netherlands, cooperation is more team-oriented and equitable, emphasizing shared decision-making and responsibility. “The department prepares for the weekly doctor’s visits by deciding together what needs to be discussed. So, it is a kind of fixed ritual. Between meetings, doctors can be contacted if a resident’s condition worsens” (Physician-NL-1). While promoting holistic care, this approach can be time-consuming and delay decisions in complex cases.

#### Record-based vs. conversation-based

A further difference between the two countries was found in the respondents’ experiences of how interprofessional collaboration is practiced.

In Germany, collaboration was mainly structured through written documentation, offering traceability but causing delays and limiting direct contact. “Yes, actually everyone should talk more to each other, but that’s just what no one has time for” (Physician-G-1). Deficient digital networking and minimal discussions contribute to delays and misunderstandings, prompting calls for improved digital solutions.

In the Netherlands, oral communication is the primary mode of interaction, occurring in regular team meetings. Additionally, conversations about residents take place informally: “We sit down with the doctors during lunch and discuss particular residents. […] The short distances, the meetings, communicating as equals, working for the same employer—that also connects us” (Nurse-NL-1). This facilitates timely coordination, shared trust and dynamic responses. However, this type of communication requires more time and resources, and the focus on conversation could reduce formal documentation.

## Discussion

This study highlights differences in medical care and interprofessional collaboration in German and Dutch nursing homes. In Germany, care heavily relies on individual initiative, often leading to isolated solutions and communication issues, whereas in the Netherlands, care is collectively organized with multidisciplinary teams. While German nursing care is more reactive, the Dutch system emphasized prevention through proactive planning to enhance quality of life. These differences are rooted in systemic structures and cultural practices unique to each country.

### Systemic differences in specialist access and coordination

Our findings reveal differences in specialist care organization and coordination. In Germany, specialist care was more accessible but also more fragmented. Nurses and family members sometimes initiate specialist consultations without the direct involvement of GPs, leading to uncoordinated care pathways. The interviewed German GPs recognized the need for neurologists and psychiatrists. In contrast, nurses perceived a much greater need for specialist involvement, which may lead to an overprovision of specialist services [23] and difficulties in coordination of medical care by GPs. The previous HOMERN study also demonstrated clear discrepancies in how different professional groups in Germany assess the need for specialist involvement in nursing homes. While GPs saw a high demand primarily for neurology and psychiatry specialists [24], they felt being able to manage other conditions without additional specialist input. Nurses, on the other hand, identified a much broader range of specialist needs [13].

By contrast, the Dutch system operates under a strong gatekeeping model, with ECPs assuming primary responsibility. Specialist consultations are not readily available on an outpatient basis. This model promotes continuity and reduces unnecessary transfers but increases ECP workload. Despite support from physician assistants and nurse practitioners, the limited number of ECPs per nursing home raises concerns about meeting all specialist needs in complex cases. Although our focus was on organization and delivery, findings suggest broader implications for residents’ wellbeing. Participants linked physician availability, communication, and team-based collaboration to residents’ safety and stability of care, showing how medical structures are interwoven with overall care experience. When interpreting these findings, it is important to note that our study focused on the prototypical Dutch model of nursing home medical care, i.e. the ECP-led system within larger nursing home organizations. This model is distinctive internationally and represents the mainstream form of institutional nursing home care in the Netherlands.

Our interview findings suggest that in Germany communication between nursing staff and GP practices is a key factor in organizing (specialist) care, rather than perceived competence. While the German system benefits from accessibility, it requires improved coordination to prevent inefficiencies. Previous survey data we collected from both countries before further contextualize these findings [18]: German nurses were more likely than Dutch nurses to see a need for specialist treatment, likely because Dutch participants experienced better communication and coordination between nursing home staff and ECPs. These results underscore the importance of structured coordination mechanisms in specialist care provision.

### Communication and interprofessional collaboration

The interviews revealed differences in interprofessional collaboration. In the Netherlands, staff described a team-oriented approach with regular multidisciplinary meetings ensuring collective decisions. The organizational culture in the Dutch working environment in general tends to be team-oriented with flatter hierarchical structures than in Germany [25]. In the nursing homes, this setup promoted shared responsibility and close communication among nurses and ECPs. A study on geriatric rehabilitation care in the Netherlands highlights that effective care for frail and multimorbid patients necessitates optimal interprofessional collaboration among various healthcare professionals [26]. The fact that all team members often share the same employer further streamlines communication and collaborative practices. In contrast, German respondents emphasized challenges in collaboration, particularly due to the limited presence of physicians in nursing homes. Many GPs in Germany are responsible for individual patients residing in multiple nursing homes, rather than being assigned to a specific facility [13]. This hinders direct communication with care staff and reducing opportunities for coordinated decision-making. Given the complexity of nursing home residents’ needs [27], this fragmented physician presence contributes to reactive rather than proactive care. A scoping review highlighted that the lack of cooperation between involved care actors in Germany, such as individuals in need of nursing care, informal caregivers, and healthcare professionals, leads to fragmented and discontinuous care [5]. Additionally, a holistic multiple case study examining intra- and interprofessional collaboration in German nursing homes identified the need for clearer role definitions, structured communication channels, and a culture of mutual respect and shared decision-making to enhance collaboration [28].

These findings raise the question to what extent the observed differences in collaboration are rooted in structural characteristics of nursing home care or reflect broader cultural patterns within the respective healthcare systems. The more hierarchical and documentation-driven collaboration reported in the German nursing homes, and the team-based, dialog-oriented approach described in the Dutch interviews, are likely shaped by both. While structural factors such as the presence of on-site physicians and shared employment models clearly play a role, professional norms, expectations toward authority, and communication styles within the broader system may reinforce or amplify these tendencies [29]. Our data do not allow us to disentangle structural from cultural influences, but suggest an interplay that deserves further investigation.

Germany could learn from the Dutch model, where reducing bureaucracy and fostering a team-based culture enhance collaboration and outcomes. To improve coordination in the German system, it would be essential to address the structural challenge of doctors being spread across several nursing homes.

### Strengths and limitations

This study offers several strengths that enhance its contribution to understanding the medical care of nursing home residents across Germany and the Netherlands. A key strength lies in the inclusion of participants from both countries and two distinct professional groups—nurses and primary care physicians (GPs and ECPs). This diversity allows for a more comprehensive exploration of cross-national perspectives and interdisciplinary insights into nursing home care. Furthermore, the interviews were conducted by native speakers, ensuring cultural and linguistic sensitivity during data collection.

However, certain limitations should be acknowledged. The sampling of nursing homes was not conducted systematically, which may introduce potential selection bias. Nonetheless, efforts were made to include facilities with varying characteristics, particularly in Germany, to capture a broad range of experiences. Small, purposively selected samples are common in qualitative research aiming to explore in-depth experiences [30], yet this approach limits the generalizability of the findings.

In the Netherlands, only one ECP was interviewed, which restricts the diversity of physician perspectives and may bias the findings toward a more favorable view of the medical care system. Recruitment was fully coordinated through our Dutch university partner, and despite repeated feedback about the need for additional ECPs, no further participants were available. The interviewed ECP may have been particularly satisfied with the system, influencing the results. However, Dutch nursing staff—who collaborate closely with ECPs—largely corroborated the views expressed in that interview, strengthening the credibility of our findings. With a larger sample of ECPs, the results might have reflected a broader spectrum of experiences.

Finally, our Dutch sample reflects the prototypical model of nursing home medical care (verpleeghuizen), where ECPs are employed by large nursing home organizations with multiple locations. While this model is widespread and represents the structural ideal of Dutch nursing home medicine, it does not cover all settings. Particularly in small-scale homelike nursing homes [31] or formerly residential homes, medical care may still be provided by GPs.

## Conclusion

This explorative study highlights key structural differences shaping interprofessional collaboration. In Germany, the lack of a defined coordination role for GPs leads to fragmented care despite easy specialist access, and structured collaboration remains a challenge. In contrast, the Dutch model fosters team-oriented care with ECP-led coordination and shared decision-making but faces issues like specialist shortages and high ECP workload.

Structural and cultural differences within healthcare systems fundamentally shape interprofessional collaboration and care coordination. Future research should examine how these systemic differences affect resident outcomes over time and compare perspectives across different facility types within countries to capture the full spectrum of medical care models.

## Data Availability

The data underlying this article cannot be shared publicly due to privacy of participants of the study. Key Points Interprofessional collaboration in nursing homes differs structurally between Germany and the Netherlands. Defined coordination roles, like ECPs in the Netherlands, support proactive, team-based care. System-level adaptations should reflect existing structures and be evaluated for impact on resident outcomes.
